# The Action of Saline Cathartics

**Published:** 1884-03

**Authors:** 


### The Action of Saline Cathartics.

Dr. Mathew Hay concludes the report of his investigation into the action of saline cathartics as follows: "They all tolerably closely agree in the ultimate effect they have on the alimentary canal and the body generally. They cause no irritation or inflammation of the canal; stimulate but in the smallest degree the secretion of the more important digestive juices, as the gastric, the pancreatic, and the biliary; have, under ordinary circum stances, little action on the blood; and mainly act by increasing the intestinal secretion, and by hindering the absorption of the intestinal fluid. Their purgative action is therefore extremely simple. They sweep out the contents of the alimentary canal with the least possible disturbance of the digestive system and of the other systems of the organism. Few other purgatives, if any, have so simple an action. The value, therefore, which has long been assigned to them in the treatment of the occasional disturbances of digestion, to which almost every one is at times subject, and where the indication seems to be to empty the canal '*cito, tut- et jucunde*,' is quite justified by the results of this investigation."–*Journal of Anatomy and Physiology.*

